# Letters from Europe

**Published:** 1846-03

**Authors:** James C. Cross

**Affiliations:** Late Professor of the Institutes of Medicine in Transylvania University


### WESTERN JOURNAL

OF

### MEDICINE AND SURGERY.

MARCH, 1846.

---

**Art. I.**—*Letters from Europe.* By James C. Cross, M. D., late Professor of the Institutes of Medicine in Transylvania University.

Paris, December, 1845.

Gentlemen:

The subjects treated of in this communication and those which may follow will not be classified, and for an obvious and, we trust, a sufficient reason. The facts as they come to our knowledge will be registered at once, and unless we were to re-write them, and for this we have not time, no particular order could be observed. If an arrangement different from that which may result from accident should be deemed necessary, it can be effected by the compositor as he sets up the several articles.

In the *Gazette des Hopitaux*, M. Rostan has some useful remarks on the *Symptomatology of Nervous Affections.* The chief phenomena to which they give rise consist in an augmentation, perversion, diminution, or abolition of the functions of sensation and motion. Formerly those of motion were apparently much more considerable and numerous than at present. The cause of this is found in the fact, that pathological anatomy has made such progress that we are now justified in referring many symptoms, considered as nervous until recently, to local lesions, such as encephalitis, meningitis, softening of the brain, &c. Some authors believe that neuralgia results exclusively from inflammation of the nerves. It is, however, generally an affection of the sensibility; we do not intend saying that nervitis never exists, but in the great majority of cases it is impossible to recognise the least appreciable trace of anatomical lesion.

Neuroses may attack the organs of all the senses; thus there may be neuroses of sight, which consists in perversion, diminution, or complete abolition of the function; neuroses of the voice; and the author speaks of a hysterical woman who was rendered completely aphonic. Bleeding, antispasmodics, blisters, and the most powerful and painful revulsives were employed ineffectually. M. Rostan felt fully assured that the woman had no motive to impose upon him; and having seen noticed in a journal another case very similar to the one under his treatment that had been completely cured by electricity, he determined to make trial of it, and to his great astonishment the voice gradually returned under its influence; from which he concludes that it was a case of nervous mutism independent of organic lesion.

The neuroses of intelligence deserve a moment's notice. On a former occasion, M. Rostan endeavored to show that every modification of mind was the result of a modification of the organ of intellection; that is to say, of some change in the nervous centres. Whenever there is a perversion of intelligence, as it cannot be admitted that an immaterial being is susceptible of perversion, we should look for the cause of the perversion in the organ of thought. It should, however, be remarked, that the existence of neuroses of intelligence is not incompatible with the principles of organicism; for why should there not be neuroses of the brain as well as other parts of the nervous system? The neuroses may attack the apparatus of organic life; allusion, however, is not made to gastralgia, for it is the most frequent and best known of them, but to those of respiration, in which there is observed nervous dyspnœa, anxiety, and difficult respiration, which with propriety can be ascribed only to a lesion of innervation. Although he has always maintained that the asthma of the aged is not a nervous disease, M. Rostan says that he never has intimated that there were not neuroses of the respiratory apparatus. Although there are cases of real nervous dyspnœa, he remarks that after having practised medicine thirty years, he has never seen a single case of what authors denominate angina pectoris, descriptions of which are so common in medical writings. With the symptoms of angina pectoris he has always found the pulsations of the heart very violent, and other signs characteristic of an organic affection of the centre of circulation, or of the lungs themselves. He regards angina pectoris as a symptom only, and maintains that all the cases of it that he has observed he could refer without difficulty to a sensible and appreciable organic alteration, but is willing to admit that others may have met with something purely nervous. He does not deny the existence of nervous pulsation of the heart, such as those caused by moral emotions, joy, sadness, fright, &c.; but all others arise from peculiar states of the blood, as in anemia, chlorosis, &c. There are neuroses of the urinary apparatus in which there are alterations of the color, consistence, &c., of the urine, and which are symptomatic of a nervous state of the secretory or excretory apparatus. He speaks of a patient who, now and then, was suddenly attacked with a very abundant mucous discharge from the nares; not only was it copious, but continuous, and resembled the white of an egg in a liquid state, and this, too, without pain, swelling of the mucous membrane, or lachrymation. Generally this happens as a consequence of some vivid moral impression. The vesical mucous membrane may be similarly affected, and then the urine presents the appearance and consistence of the catarrhal urine, with which physicians are familiar. Neuroses of the genital organs are very common. Priapism, satyriasis, and nymphomania, are doubtless purely nervous, and wholly independent of all material lesion. He professes not to know exactly the seat of these affections, but is inclined to refer them, with Gall, to the brain, as that organ is the admitted seat of hysteria. This is but a brief and rapid outline of the views of M. Rostan, on the principal neuroses, and we shall add but a few words in regard to what he says on the subject of their diagnosis. They are uniformly intermittent, and this is a most important circumstance in doubtful cases. When they are continuous they are exceedingly difficult to recognise, and great skill and experience are necessary to determine the diagnosis with any degree of precision. The neuroses are usually very protracted; they often persist for months, and sometimes even for years. They rarely terminate in death, and the prognosis, if deduced from the intensity of the symptoms, would bespeak greater danger than exists in reality. The neuroses after having continued a certain length of time become chronic, and then they are rarely cured; and when this happens, as it does sometimes, we are surprised at the manner in which it is effected; it takes place suddenly and apparently without cause. In no other affection has hereditariness, as a cause, more influence than in the neuroses. It is very common to witness them in those persons whose parents have been similarly affected.

*Spontaneous Cure of Phthisis-Pulmonalis.*—It is not uncommon to find, in post mortem examinations, puckered depressions, generally at the summits of the lungs, which are considered as the result of ancient cicatrices in those who have not fallen victims to pulmonary consumption. Dr. Bennett, it appears, has recently been prosecuting the investigation of this subject in the Royal Infirmary of Edinburgh, and he found old cicatrices of the lungs in 28 cases out of 73; and this result added to that obtained by M. M. Rogee and Boudet of this city—for they have been inquiring into the matter also—

establishes the fact that phthisis has been spontaneously cured in one-third, if not one-half, of those who die after the age of forty. This result, which is opposed to the common opinion, and which reposes upon facts that it is impossible to attribute to any other cause, is not, however, contradicted by what we know of the chemical and organic composition of tubercles, or with what we have been taught by the study of their development. Indeed, all that we certainly know of the chemical composition of tubercle, is reduced to the established fact that in the first period of its development it differs from lymph only because it contains more albumen, and in its latter period more earthy salts. As to its intimate organization, it is certain that it is not of the nature of the malignant tissues, cancerous for example; and notwithstanding the opinion of M. M. Gulliver and Vogel, who contend that it is not organized, we can detect traces of cellular organization, but much more numerous granulations and variously formed corpuscles that cannot be easily described, but which are readily recognised when they have been once distinctly observed. Two opinions prevail as to the formation of tubercles. Some ascribe them to inflammation, and others to a peculiar and vitiated state of the constitution. Now, neither of these are irreconcileable with the idea that phthisis may be spontaneously cured. The only difference, in truth, between tubercle and the products of ordinary inflammation consists in the absence in the former of all disposition to become organized; tubercle presents granulations and imperfect cells, while in the products of normal inflammation all the elements are perfect. Now, as these two different products are formed by the exudation of the *plasma* of the blood, the essential distinction between them should be found in a difference in the composition (chemical and vital) of the *plasma* of the blood that enters into their composition. Hitherto chemistry has reflected no light upon the exact nature of this difference, but it is probable that it results from the presence of *protein*, which has not so great a tendency to become organized as fibrin, and it is quite certain that when tuberculous matter is reduced to the molecular state by disintegration, it may be as readily observed as the products of normal inflammation. If, therefore, there is nothing in the nature of the elements of tubercle to prevent their absorption, there is no reason why we should refuse to regard the cicatrices found most generally in the summits of the lungs, where we know that the tubercles are most commonly deposited, and when, too, they are discovered in aged persons who have died of other diseases, as evidence of the previous existence of phthisis pulmonalis, and of its spontaneous cure.

The treatment of tubercular consumption has heretofore been almost exclusively directed on empirical principles, and consequently no one has furnished absolutely useful results. Perhaps it would have been otherwise had we observed the course pursued by nature in the cures that have been spontaneously effected. This we have endeavored to do, and, we think, not without encouraging results. But two indications need be observed in the management of those threatened with, or actually laboring under phthisis. First, the morbid state of the blood that results from imperfect nutrition; and secondly, the local inflammation that produces an abnormal secretion—a secretion containing the elements of tubercle. These are the indications which Dr. Bennett points out, and to which he invites the particular attention of physicians; but if the reader will take the trouble to refer to the *Western and Southern Medical Recorder*, he will find them laid down by the editor, and dwelt on in a sufficiently full and explicit manner. All the information we have on this subject—derived from the researches of chemistry, morphology, and physiology—go to show that the state of the blood, in the first place, is mainly attributable to an excess of oxygen in the economy, which combines with the tissues—causes their destruction, and produces acidity of the alimentary canal, and afterwards to an excess of azotized or albuminous matters, and at the same time to the absence of carbon and oleaginous matters in the chyle, the blood, and other tissues, with the exception of the liver, which is the great emunctory of fatty and carbonaceous matters. There are then three objects to be kept constantly in view in the treatment of phthisis. First, to remedy the dyspeptic state of the alimentary canal, and particularly the acidity of it which too frequently abounds; secondly, to direct such articles of food to be eaten as promise to form a suitable kind of chyme; and lastly, to subdue the local inflammation. There are various means for the first purpose, but Dr. Bennett particularly commends mixtures with which he has perfectly succeeded in some cases, and with which he has subdued vomiting that had triumphantly resisted all other modes of treatment; the second is to be attained by a proper regimen: digestible food, milk, oleaginous and albuminous substances, and an equable climate, the tendency of which should be to diminish the excess of oxygen; of these means, Dr. Bennett particularly recommends cod-liver oil; and the third should be counteracted and controled by local blood-letting—cups are preferable to leeches. Now, those who are familiar with what we have taught, or with the contents of the *Western and Southern Medical Recorder*, know full well that the above principles of treatment have been inculcated in the most emphatic manner for a number of years, and this course was pursued, not only because of the great advantages of an invigorating over a debilitant treatment, but because the latter has been recommended by a teacher more notorious than distinguished, and who has exerted a more extensive influence over the destinies of the profession in the valley of the Mississippi than any man of ten times his merit should. His *diet absolute* has immolated thousands, and we think it full time that the public should be cured of its credulity, and he of his infatuation.

*Successful Operation for Cataract after sixty years of Blindness.* By M. Serres, Professor of the Faculty of Montpelier.—Boichat, 67 years of age, cutler by trade, consulted M. Serres in March, 1844, for a wound of the right eye, which had been several days previously inflicted by a foil. Treatment failed to prevent a loss of the sight of the wounded eye.

which reduced the patient to a state of perfect blindnes, for he had lost the sight of the other sixty years before from small-pox. The patient desiring M. Serres to operate on the right eye for cataract, he was induced to examine the left, in which he recognized the existence of a lenticular cataract, together with a slight albugo upon the external lateral segment of the cornea. Believing that the operation, if performed on the right eye, would be embarrassed with many difficulties, he proposed to operate on the left. To this the patient submitted, although under the conviction that it would prove useless, inasmuch as it had been of no service to him for the space of sixty years. A principal motive of M. Serres for wishing to operate on the left eye was to ascertain, if possible, how long the function of an organ may be suspended without being annihilated. The operation was performed, and even before it was finished, the patient exclaimed that he could see. This was believed at the time to be an optical illusion, but three days afterwards when the patient was visited for the purpose of ascertaining if there were any hope of the success of the operation, M. Serres was greatly surprised to find that it was perfect, and that the sight of the left eye was completely restored. Eighteen months after the operation the patient was still in the enjoyment of the vision of the left eye. This fact, which is perhaps unique in its kind, is a triumphant refutation of the assertion made by many writers, that in very ancient cataracts an operation must prove unproductive of good, as there is reason to believe that when the retina has been for a great length of time condemned to repose it has lost its sensibility. It cannot but be regarded as a very curious and interesting fact that after sixty years of a total suspension of its function, the faculty of distinguishing objects should be recovered simultaneously with the impinging of light upon the retina. It should be remembered that the vision of Boichat is not of that feeble and vague sort which is scarcely sufficient to conduct one along a street, but of that clear and full kind which enables him to read with facility. It may be objected that in this case, as the cataract was incomplete, the retina had never been entirely secluded from the contact of the light. To this, Mr. Serres responds, that the man assured him in the most positive and formal manner that he had been completely blind of the left eye ever since he was six years of age—that after the loss of the right and before the left was operated on, he was entirely blind, and besides, at the moment of the operation the opacity of the pupil was so complete, that had not allowance been made for this circumstance the immobility of the iris would have led to the belief that the cataract was complicated with amaurosis. Had the integrity of the visual functions of the right side any agency in preserving the sensibility of the left? This is very possible, and therefore a difference may exist between those persons who have lost the sight of both eyes and those who have lost that of one only. As it is now evident that the sensibility of the retina is retained, notwithstanding the indefinite suspension of its functions, would it not be discreet when both eyes are affected with cataract, not to operate on both at the same time? If but one should be operated on, the other could be at some subsequent period, in the event of its failure, or in the event of the sight of it being lost after it had been restored?

*Tobacco in Facial Neuralgia.*—M. Gower commends in the strongest terms the use of tobacco in neuralgia of the face. For twenty years he has employed the tincture and infusion, locally applied, with the greatest success. This success, however, he remarks, was in some degree unmerited, for although his success was decided and satisfactory, he did not know whether it was owing to the stimulant or sedative action of the tobacco. He continued its empirical employment until M. Chippendale demonstrated that its efficacy was owing to the narcotine, and that the essential oil of the plant retarded its action by preventing the absorption of its narcotic principles. It is therefore to the extract of narcotine that the preference should be given. M. Gower has seen in three cases a single application of the aqueous solution of this extract, almost instantly not only calm, but prevent the recurrence of neuralgic paroxysms. This preparation rubbed on the side of the face is said also to relieve, almost instantly, tooth-ache.

*Beef-Gall in Constipation.*—Dr. Allnat remarks that although beef-gall is not at present employed by physicians, it does not merit the desuetude into which it has fallen. He speaks of it in constipation in the most eulogistic terms. His opinion is deduced from two classes of facts—the one theoretical—the other practical. Habitual constipation, he remarks, in sedentary persons, commonly depends upon the absence of bile. The contents of the bowels pass slowly through them because they are not sufficiently acted on by their natural stimulus to produce the degree of peristaltic action necessary to cause the evacuation of their contents. When this happens the stools are of a greyish complexion and solid, because of the absorption of the more liquid parts, and they then collect in masses in the large intestines, and assume a globular form, obstructing the bowels mechanically, and thus preventing the evacuation of their contents. Now, the author asserts, if these consolidated masses are exposed, out of the body, to the action of beef-gall, they immediately swell and are in a short time dissolved and present the appearance of natural fecal matter in color and consistence. The same effect is produced if beef-gall diluted with water is injected into the bowels of an individual laboring under obstinate constipation. Three cases, which we shall not trouble you with, are adduced in support of this assertion, and in which, after the employment of the most active purgatives and the most irritating injections, a clyster of beef-gall or the administration of pills made of the bile, after its evaporation and reduction, to the consistence of an extract, produced without griping or any other inconvenience, natural stools.

Now, besides the fact which we have already shown, and we trust in a satisfactory manner, that the paristaltic action of the bowels does not depend upon the supposed stimulant properties of bile, and upon which the whole of Dr. Allnatt's theory rests; the bile has other and more elevated purposes in the animal economy. That it is an excrementitial secretion is now no longer a matter of doubt, and that its suspension interferes directly with nutrition, and produces general and very various derangements, we have illustrated and proved, and therefore if beef-gall has any particular efficacy in removing constipation dependent upon deficient or perverted action of the liver, or indeed, in the cure of any affection resulting from a kindred cause, it must be upon some other principle than that assumed by Dr. Allnat. Besides the theoretical absurdity into which the advocate of beef-gall has involved himself, the practical interest of his article rests upon one fact which he has failed to establish, but which is indispensable. He alleges that dry and hard feces are dissolved by beef-gall; that may be, but he has not shown us that it accomplished that object any better than oil or other fatty substances would under similar circumstances. When this fact is once established we may be encouraged to inquire whether or not it be true that the bile of one animal, and formed of very different materials, can perform the office of bile in the economy of another, not only of a different species, but different genus. The difference in the bile of herbivorous and carnivorous animals is amongst the most familiar of physiological facts.

Amongst the virtues of beef-gall there is one signalized by Dr. Allnatt, a notice of which should not be omitted entirely. We allude to its action upon opium with which it is combined, and the narcotic property of which it destroys. The constipating effect of opium is ascribed to its action on the liver, the secretion of which it either suspends or so much diminishes as to be insufficient to stimulate the bowels. This is a formidable objection to opium when we desire to produce its sedative effects only. If Dr. Allnat is to be believed, beef-gall is effectual in neutralizing the constipating effects of opium without impairing in the least its sedative action.

